# Evaluating the psychometric properties of an e-based version of the 39-item Parkinson’s Disease Questionnaire

**DOI:** 10.1186/s12955-014-0193-1

**Published:** 2015-01-23

**Authors:** David Morley, Sarah Dummett, Laura Kelly, Jill Dawson, Crispin Jenkinson

**Affiliations:** Health Services Research Unit, Nuffield Department of Population Health, University of Oxford, Old Road Campus, Oxford, OX3 7LF UK

**Keywords:** PDQ-39, Parkinson’s disease questionnaire, e-based, ePDQ, Electronic patient reported outcomes, ePROs, Reliability, Validity

## Abstract

**Background:**

The 39-item Parkinson’s Disease Questionnaire (PDQ-39) is the most thoroughly validated and extensively used self-report measure for the assessment of health-related quality of life in people with Parkinson’s (PwP). Given the extent of its use and increasing emphasis on electronic data capture, an e-based version of the PDQ-39, the ePDQ, has recently been developed. The aim of this short report is to present some key reliability and validity data that confirm the psychometric quality of the ePDQ.

**Findings:**

Participants were emailed a unique link to an online survey incorporating the ePDQ and demographic questions. A total of 118 PwP fully completed the survey. Floor and ceiling effects were calculated to ensure responses were not biased to extreme values. Consequently, score reliability was assessed by item-total correlations with a range from 0.34 to 0.90. Cronbach’s alpha was calculated at between 0.64 and 0.95 for the eight domains of the ePDQ. Construct validity was assessed by comparing domain scores in relation to disease duration and gender, with hypothesised differences being largely confirmed. Construct validity was further assessed following a higher order factor analysis which confirmed the appropriateness of calculating a summary index score. Subsequently, significant, but moderate correlations were calculated between the ePDQ summary index score and disease duration and age at diagnosis.

**Conclusions:**

Results indicate that the ePDQ largely mirrors the properties of its parent instrument, the PDQ-39, in terms of reliability and validity. Potential users can therefore incorporate the ePDQ into computer-based data capture systems with confidence.

## Introduction

The 39-item Parkinson’s Disease Questionnaire (PDQ-39) [1] is the most thoroughly validated and extensively used self-report measure for the assessment of health-related quality of life in people with Parkinson’s (PwP) [2]. The measure has been shown to possess sound psychometric properties [1,3-6] and its use is widely recommended [2,7,8]. Given the extent of its use and the increasing emphasis on electronic data capture [9-12], an electronic version of the PDQ-39, the ePDQ, has recently been developed. The acceptability and usability of the ePDQ have previously been reported, alongside a study assessing the impact of implementing non-response options versus ‘forced response’ on response rates and data completeness [13].

It has been suggested that full-scale psychometric analysis is not strictly necessary when developing electronic patient reported outcomes (ePROs) which closely mirror their paper-based equivalent [14]. However, given the extensive use of the PDQ-39, potential users of the electronic version may require reassurance that its psychometric properties are consistent with those of the paper-based version. The aim, therefore, of this short report is to present some key reliability and validity data that confirm the psychometric quality of the ePDQ.

## Materials and methods

Ethical approval for the study was granted by the Medical Sciences Inter Divisional Research Ethics Committee of the University of Oxford (reference MSD-IDREC-C1-2013-17).

### Participants

Recruitment of participants was undertaken with the support of Parkinson’s UK. Study details were distributed by the charity’s Research Communications Office to their research support network across the United Kingdom. Potential participants were requested to contact the research team by telephone or email.

### Materials

The ePDQ [13], an electronically administered version of the PDQ-39 [1] was administered using Qualtrics survey software [15]. The measure totals 39 items, assessing eight domains of health; Mobility, Activities of Daily Living, Emotional Well-Being, Stigma, Social Support, Cognitions, Communication and Bodily Discomfort. Domain scores are coded on a scale of 0 (perfect health as assessed by the measure) to 100 (worst health as assessed by the measure). A summary index score can subsequently be calculated from the eight domains outlined above [16]. Demographic data (gender, age, marital status, ethnic origin and age at or year of diagnosis) were obtained after participants had completed the ePDQ items.

### Procedure

After contacting the research team, participants were emailed a unique link to the online survey which could be completed in their own time and at a location of their choice (e.g. home, workplace). A follow-up email was sent after two weeks to non-responders.

### Statistical analysis

Data was checked for normality of distribution and presence of outliers prior to statistical analysis. Floor and ceiling effects were calculated for each domain of the ePDQ. Reliability was assessed via corrected item-total correlations and Cronbach’s alpha. Validity was determined through calculation of differences between known groups using independent samples t-tests. Higher order factor analysis was used to create a summary index score. Relationships between the summary index score and relevant demographic variables were tested via Pearson correlations coefficients in order to further assess validity.

## Results

A total of 118 PwP (66 males, 52 females) fully completed the ePDQ, a response rate of 91.4%. The mean age was 63.48 years (standard deviation (SD) 8.66), mean disease duration 5.73 years (SD 4.34) and mean age at diagnosis 57.69 years (SD 9.00).

### Reliability

Floor and ceiling effects were calculated to ensure responses were not biased to extreme values (see Table 1). Consequently score reliability was assessed by item-total correlations (also reported in Table 1) with a range from 0.34 to 0.90. Cronbach’s alpha was calculated at between 0.64 and 0.95 for the eight domains of the ePDQ, indicating sufficient to excellent internal reliability. Alpha values for all domains can again be viewed in Table 1.

**Table 1:** **ePDQ item-total correlations with domain floor and ceiling effects and Cronbach’s alpha values**

**Domain**	**Item no**	**Item**	**Item-total correlation (corrected)**	**% Floor**	**% Ceiling**	**α**
***Mobility***				1.7	4.2	0.95
	1	Had difficulty doing leisure activities	0.69			
	2	Had difficulty looking after your home	0.76			
	3	Had difficulty carrying bags of shopping	0.73			
	4	Had problems walking half a mile	0.86			
	5	Had problems walking 100 yards	0.87			
	6	Had problems getting around the house	0.84			
	7	Had difficulty getting around in public places	0.90			
	8	Needed to be accompanied when out	0.78			
	9	Frightened or worried about falling in public	0.67			
	10	Been confined to the house more than liked	0.82			
***Activities of daily living***				0.8	1.7	0.88
	11	Had difficulty washing yourself	0.72			
	12	Had difficulty dressing yourself	0.80			
	13	Had problems doing up buttons or laces	0.78			
	14	Had problems writing clearly	0.49			
	15	Had difficulty cutting up food	0.80			
	16	Had difficulty holding a drink	0.65			
***Emotional well-being***				0.8	5.9	0.88
	17	Felt depressed	0.71			
	18	Felt isolated and lonely	0.80			
	19	Felt weepy or tearful	0.72			
	20	Felt angry or bitter	0.67			
	21	Felt anxious	0.63			
	22	Felt worried about the future	0.68			
***Stigma***				0.8	22.0	0.83
	23	Felt you had to conceal PD from people	0.57			
	24	Avoided eating or drinking in public	0.54			
	25	Felt embarrassed by having PD	0.80			
	26	Felt worried by other’s reaction to you	0.73			
***Social support***				0.8	39.8	0.76
	27	Had problems with close relationships	0.58			
	28	Not had support from spouse or partner	0.66			
	29	Not had support from friends or family	0.57			
***Cognitive impairment***				0.8	1.7	0.64
	30	Unexpectedly fallen asleep during day	0.38			
	31	Had problems with concentration	0.59			
	32	Felt your memory was bad	0.40			
	33	Had distressing dreams or hallucinations	0.35			
***Communication***				0.8	21.2	0.87
	34	Had difficulty with speech	0.78			
	35	Felt unable to communicate properly	0.82			
	36	Felt ignored by people	0.67			
***Bodily discomfort***				0.8	6.8	0.71
	37	Had painful muscle cramps or spasms	0.60			
	38	Had aches and pains	0.58			
	39	Felt unpleasantly hot or cold	0.41			

### Validity

Construct validity was assessed by comparing domain scores in relation to disease duration (groups split based on median disease duration of 4 years) and gender, as seen in Table 2. All domains anticipated as being significantly different for disease duration were confirmed as such; Mobility; Activities of Daily Living; Cognitive Impairment; Communication; Bodily Discomfort. Hypothesised non-significant differences between males and females were confirmed in all but one of the eight ePDQ domains. Construct validity was further assessed following a higher order factor analysis which confirmed the appropriateness of calculating a summary index score. One factor with an Eigenvalue in excess of one was identified, accounting for 55.4% of variance (extraction method principal component analysis). Each domain of the ePDQ loaded on this one factor which had an eigenvalue of 4.4. Subsequently, all eight domains of the ePDQ were summed to create the summary index score. The mean value of the summary index was 28.67 (S.D. = 16.66, min = 2.97, max = 91.93). Finally, validity was further established by significant, but moderate correlations between the ePDQ summary index score and disease duration (r = .27, p < 0.01) and age at diagnosis (r = −.27, p < 0.01).

**Table 2:** **Comparison of ePDQ domains by disease duration and gender**

	**Disease duration**			**Gender**		
	**1-4 years**	**5-20 years**	***p***	**Male**	**Female**	***p***
	**(n = 59)**	**(n = 59)**		**(n = 66)**	**(n = 52)**	
Mobility	27.03 *(26.87)*	41.27 *(25.47)*	<.00	31.86 *(25.59)*	37.07 *(28.73)*	NS
Activities of daily living	28.11 *(21.86)*	39.62 *(24.15)*	<.00	33.58 *(23.01)*	34.21 *(24.67)*	NS
Emotional well-being	26.20 *(21.16)*	32.06 *(20.10)*	NS	26.83 *(20.87)*	32.05 *(20.45)*	NS
Stigma	17.58 *(19.37)*	24.68 *(23.31)*	NS	19.51 *(21.19)*	23.20 *(22.22)*	NS
Social support	14.12 *(21.84)*	15.34 *(17.12)*	NS	17.30 *(21.45)*	11.70 *(16.52)*	NS
Cognitive impairment	25.53 *(18.29)*	32.84 *(17.96)*	<.05	29.45 *(17.36)*	28.85 *(19.85)*	NS
Communication	22.88 *(23.14)*	33.05 *(25.85)*	<.05	32.07 *(23.03)*	22.76 *(26.51)*	<.05
Bodily discomfort	35.17 *(23.67)*	47.17 *(24.05)*	<.00	38.13 *(24.98)*	45.03 *(23.58)*	NS

(*standard deviation;* NS = non-significant).

## Discussion

This short report has presented a brief analysis of the newly developed ePDQ as a means of confirming that the measure reflects qualities that are consistent with the paper-based PDQ-39 [1]. Floor and ceiling effects generally mirror those of the parent instrument, largely falling within previously used criteria of 20% [17]. It is acknowledged, however, that the domain of Social Support is almost double this figure, which may be a reflection of the inherent weakness of three-item domains. Reliability of the ePDQ is confirmed by two commonly used analyses, item-total correlations and Cronbach’s alpha values. All item-total correlations are in excess of previously defined criteria [18], the majority significantly so, thereby confirming that item scores within each domain are related to the overall domain score and thus, to the underlying construct. Cronbach’s alpha coefficients are above the recommended level of 0.70 in all but one domain, indicating good to excellent internal reliability [19]. The one domain to fall below this figure, Cognitive Impairment at 0.64, remains above the level regarded as sufficient [20].

The validity of the ePDQ was confirmed via a number of analyses. Firstly, a comparison of known groups was made, (sometimes referred to as known groups validity). Such an assessment is made where there are good reasons to hypothesise that scores on a construct being measured will differ between two groups [21], as has been addressed in previous research [22,23]. When comparing domains by disease duration those hypothesised as being significantly different were confirmed (Mobility, Activities of Daily Living, Cognitive Impairment, Communication and Bodily Discomfort). The three domains of Emotional Well-Being, Stigma and Social Support are deemed not to be as sensitive to disease duration with results appearing to support this. Additionally, non-significant differences between males and females were observed in seven of the eight ePDQ domains. Construct validity was further supported through the use of a higher order factor analysis to confirm the appropriateness of summing domain scores to create a summary index score. This technique has been widely used in previous research [16,24-27]. Summary index scores were found to correlate significantly, and in the anticipated direction, with relevant demographic variables to provide further evidence of validity.

It is acknowledged that this study may be limited by the relatively small sample size, although this is adequate for the analyses reported. It is, however, recommended that the ePDQ be further assessed in larger surveys and over time in longitudinal studies. Assessment of the sensitivity of the ePDQ is also required, although given that the reliability and validity of the measure largely mirrors that of the paper-based version, it is anticipated that sensitivity data should follow a similar pattern. Additionally it is acknowledged that the sample reported here may not be entirely representative of the Parkinson’s population at large, as not all PwP will have access to electronically administered measures or be computer literate.

In conclusion, data indicates that the ePDQ possesses appropriate levels of reliability and validity and can be incorporated into studies with confidence by those who wish to do so. Should readers wish to make direct comparisons between the psychometric properties of the PDQ-39 and ePDQ they can do so via the data presented here and the original validation paper for the PDQ-39 [1] or current user manual [6]. Further details of the ePDQ, the PDQ-39 and other related measures, along with how to obtain copies, can be obtained from DM or CJ.

## Abbreviations

ePDQ: Electronic Parkinson’s Disease Questionnaire
ePROs: Electronic patient reported outcomes
PDQ39: 39-Item Parkinson’s Disease Questionnaire
PwP: People with Parkinson’s
SD: Standard deviation
